# Impact of Genetic Variants on Pregabalin Pharmacokinetics and Safety

**DOI:** 10.3390/ph18020151

**Published:** 2025-01-23

**Authors:** Sofía Calleja, Andrea Rodríguez-López, Dolores Ochoa, Sergio Luquero, Marcos Navares-Gómez, Manuel Román, Gina Mejia-Abril, Samuel Martín-Vilchez, Francisco Abad-Santos, Pablo Zubiaur

**Affiliations:** 1 Clinical Pharmacology Department, Hospital Universitario de La Princesa, Faculty of Medicine, Universidad Autónoma de Madrid (UAM), Instituto de Investigación Sanitaria La Princesa (IIS-Princesa), 28006 Madrid, Spain; 2 Centro de Investigación Biomédica en Red de Enfermedades Hepáticas y Digestivas (CIBERehd), Instituto de Salud Carlos III, 28029 Madrid, Spain

**Keywords:** pregabalin, pharmacokinetics, safety, pharmacogenetics

## Abstract

**Background/Objectives:** Pregabalin is a useful therapeutic option for patients with anxiety or neuropathic pain. Genetic variants in certain genes encoding for transporters related to absorption and distribution could have an impact on the efficacy and safety of the drug. Furthermore, extreme phenotypes in metabolic enzymes could alter pregabalin-limited metabolism. **Methods**: In this study, we included 24 healthy volunteers participating in a bioequivalence clinical trial and administered pregabalin 300 mg orally; 23 subjects were genotyped for 114 variants in 31 candidate genes, and we explored their impact on pregabalin pharmacokinetics and safety. **Results**: The uncorrected mean (±SD) of AUC_∞_ and C_max_ were 61,097 ± 14,762 ng*h/mL and 7802 ± 1659 ng/mL, respectively, which were significantly higher in females than in males (*p* = 0.002 and *p* = 0.001, respectively), with no differences in dose/weight (DW)- corrected exposure metrics. NAT2 slow acetylators (SAs) showed a 16–18% increase in exposure compared to intermediate (IAs) and normal (NAs) acetylators; NAT2 SAs exhibited a 25% higher t_1/2_ as compared with NAT2 IAs and 58% higher compared to NAT2 NAs. In contrast, neither the NAT2 phenotype nor other genetic variants were related to pregabalin adverse drug reaction (ADR) occurrence. On the contrary, sex and sex-related exposure differences (i.e., females and their higher exposure compared to males) were the main predictors of ADR occurrence. **Conclusions**: Our findings suggest that NAT2 could be partially responsible for the minor proportion of pregabalin metabolism, but the effect of NAT2 phenotype does not seem clinically relevant. Therefore, pharmacogenetic biomarkers appear to play a restrained role in pregabalin pharmacotherapy.

## 1. Introduction

Pregabalin is similar in structure to the endogenous neurotransmitter gamma-amino butyric acid (GABA) [1]. GABA, a non-proteinogenic amino acid released by interneurons, serves as the major inhibitory neurotransmitter in the central nervous system (CNS), regulating the excitability of neuronal networks by binding to GABA_A_ [2] and GABA_B_ [1,3] receptors located on postsynaptic neurons distributed in the central nervous system. Although its exact mechanism of action has not yet been fully characterized, pregabalin does not bind to GABA receptors but to α2-δ protein, an auxiliary subunit of voltage-dependent calcium channels in the central nervous system, which affects the entry of calcium into nerve cells [4]. This reduces the activity of certain neurons in the brain and spinal cord, decreasing the release of neurotransmitters involved in neuropathic pain, epilepsy, postherpetic neuralgia, fibromyalgia, and generalized anxiety disorder.

The maximum recommended dose by the Food and Drug Administration (FDA) is 100 mg three times a day (300 mg/day). In contrast, the European Medicines Agency (EMA) drug label recommends 150 to 600 mg daily [1,5]. After oral administration, pregabalin is highly absorbed, with a bioavailability ≥ 90% at the therapeutic doses [6]. Pregabalin exhibits linear pharmacokinetics over the recommended daily dose range. Therefore, pregabalin therapeutic drug monitoring (TDM) is not necessary [7,8]. Maximum plasma concentrations (C_max_) are observed 1 h after oral administration (t_max_). Food reduces pregabalin C_max_ by approximately 25% to 30% and increases the t_max_ to approximately 3 h, yet the total amount of drug absorbed is unaltered [1,5]. The area under the time-concentration curve (AUC) from time zero to infinity (AUC_∞_) with the 150 mg dose is 27,904.24 ng*h/mL, and the C_max_ is 3849.50 ng/mL [9]. The apparent volume of distribution (V_d_) following oral administration is approximately 0.5 L/kg. Pregabalin does not bind to plasma proteins and the mean terminal half-life (t_1/2_) is approximately 6.3 h in subjects with normal renal function. Only a minor fraction of pregabalin is metabolized, as it is primarily eliminated by renal excretion unaltered. Dose adjustments in adults with reduced renal function are required [1,5,9]. However, the claim in the U.S. and European drug labels that pregabalin undergoes no more than 2% metabolism is based on mass balance studies [1,5] conducted in healthy volunteers. This same assertion is commonly repeated in the literature [10], and even creatinine clearance (CrCl) has been equated with pregabalin clearance [11]. However, the studies were conducted in healthy volunteers, and it therefore cannot be ruled out that in patients with impaired renal function, unknown metabolic pathways may occur.

Pregabalin is a useful therapeutic option for patients with anxiety or neuropathic pain [12,13]. Genetic variants in certain genes encoding for transporters related to absorption, distribution, and permeation into the CNS across the blood-brain barrier (BBB) could have an impact on the efficacy and safety of the drug. Similarly, it is possible that variants in metabolizing enzymes, such as cytochrome P45O (CYP) isoforms, may have an impact on drug exposure. Although the fraction of pregabalin that is metabolized is very low c, and to our understanding, no CYP or UGT enzymes have been reported to metabolize pregabalin to date, patients with “extreme” pharmacogenetic phenotypes, such as ultrarapid metabolizers (UM) for CYP2D6 or CYP2C19, or poor metabolizers (PM) for CYP2C9 or CYP2B6, among others, could suffer unexpected alterations in metabolism. If these extreme phenotypes occurred in patients with impaired renal function, the impact could be even larger. Therefore, the aim of this study was to evaluate the impact of 114 variants in 31 relevant pharmacogenes on pregabalin pharmacokinetic variability and tolerability. This broad genotyping approach has been conducted on numerous occasions in our laboratory. It allows for the continued characterization of pharmacokinetics in the post-marketing clinical context, including antiepileptic drugs and other CNS agents [14,15,16,17,18,19]. The observation of associations enables the generation of hypotheses that provide a broad and complementary perspective to studies carried out during the drug development phase, where certain limitations arise, such as the use of healthy volunteers to exclusively characterize pharmacokinetics.

## 2. Results

A total of 24 healthy volunteers participated in the study, but one female did not consent to genotyping. The mean age was 34 ± 10 years old, with evenly distributed proportions of males (n = 10) and females (n = 14); there were 3 Europeans and 21 healthy volunteers with Mixed race (i.e., 20 Latin Americans and 1 Sub-Saharan African). The Europeans showed higher height (*p* = 0.030) than the healthy volunteers with Mixed race (Table 1). Females exhibited lower height (*p* < 0.010) than males, lower weight (*p* < 0.010), and lower body mass index (BMI) (*p* = 0.037) (Table 1).

### 2.1. Pharmacokinetics

The uncorrected mean (SD) of AUC_∞_ and C_max_ were 61,096.81 ± 14,762.24 ng*h/mL and 7801.99 ± 1658.61 ng/mL, respectively. For females, the uncorrected mean AUC_∞_ was 67,981.98 ± 14,290.93 ng*h/mL compared to 51,457.56 ± 9216.02 ng*h/mL for males (*p* = 0.002). For the uncorrected C_max,_ these values were 8653.39 ± 1570.09 ng/mL and 6610.04 ± 873.67 ng/mL, respectively (*p* = 0.001). However, no differences in dose/weight (DW)-corrected AUC or C_max_ were observed according to sex (Table 2). In contrast, males showed higher V_d_/Fw than females (univariate *p* = 0.001; unstandardized beta coefficient [β] = 0.180, multivariate *p* = 0.003, R^2^ = 0.36) and a higher t_1/2_ (β = 0.186, R^2^ = 0.598, *p* < 0.001) (Table 2). No differences were found between European and Mixed race.

Significant associations between polymorphisms and the pharmacokinetic parameters of pregabalin are shown in Table 3. Subjects with *UGT1A6* rs7592281 G/T genotype or with the NAT2 SA (slow acetylator) phenotype showed higher AUC/DW than those with the *UGT1A6* rs7592281 G/G genotype (univariate *p* = 0.007) or NAT2 RA (rapid acetylator) plus IA (intermediate acetylator) phenotypes (univariate *p* = 0.042; β = 0.185, multivariate *p* = 0.022, R^2^ = 0.236), respectively. Consistently, NAT2 SAs showed higher t_1/2_ than IAs (univariate *p* = 0.05) and RAs (univariate *p* = 0.002) and both combined (β = 0.196, multivariate *p* < 0.001, R^2^ = 0.598) and lower Cl/Fw (β = −0.183, multivariate *p* = 0.023, R^2^ = 0.36).

Volunteers with CYP2B6 rapid metabolizer (RM) and normal metabolizer (NM) phenotypes showed higher t_1/2_ than volunteers with intermediate metabolizer (IM) and PM phenotypes (univariate *p* values: 0.026 and 0.002, respectively) (Table 3).

Subjects with *CES1* rs8192935 C/C genotype showed higher C_max_/DW compared to those with the T/C genotype (univariate *p* = 0.018) and with those with T/C+T/T genotypes combined (β = 0.185, multivariate *p* < 0.001, R^2^ = 0.548). Moreover, subjects with CYP4F2 *1/*5 diplotype showed lower C_max_/DW than those with CYP4F2 *1/*3 or *1/*4 diplotypes (univariate p=0.013)*,* and those carrying CYP4F2 *1/*1 or *1/*5 diplotypes showed lower C_max_/DW when compared with the rest of diplotypes (β = −0.167, multivariate *p =* 0.001, R^2^ = 0.548). *UGT1A6* rs7592281 T allele carriers showed higher C_max_/DW than those with the G/G genotype (univariate *p* = 0.006) (Table 3). Finally, CYP3A5 IMs showed higher t_max_ than PMs (univariate *p* = 0.020; β = −0.464, multivariate *p* = 0.003, R^2^ = 0.36). No statistically significant differences in genotype or phenotype prevalence were observed according to the biogeographic origin.

### 2.2. Safety

A total of 38 adverse reactions were reported between the two formulations of pregabalin. These reactions were experienced by 19 of 24 volunteers; 58.3% of the volunteers suffered from dizziness, this being the most common adverse drug reaction (ADR). Of all volunteers, 75% suffered CNS symptoms, 20.8% gastrointestinal symptoms, and 8.3% musculoskeletal ADRs (MSADRs).

Patients showing any ADR showed a statistically significant higher AUC than those that showed no ADR (64,645.24 ± 14,111.85 ng*h/mL vs. 47,612.77 ± 8356.13 ng*h/mL, *p* = 0.007) and C_max_ (8202.66 ± 1550.93 ng/mL vs. 6256.68 ± 1106.11 ng/mL, *p* = 0.009). The same finding was observed for dizziness occurrence and CNS symptoms (*p* < 0.05).

Women showed a higher prevalence of ADRs of any type than men and higher rates of CNS ADRs. Volunteers with *ABCB1* rs2032582 G/G genotype showed a higher incidence of any type of ADR compared to those with G/T and T/T genotypes (100% vs. 83.3% and 40%, respectively). Individuals with *CES1* rs2244613 C/C genotype, *CES1* rs8192935 T/T or *SLC19A1* rs1051266 A/A genotypes showed higher incidence of gastrointestinal symptoms (GI) compared to those with *CES1* rs2244613 C/A or A/A genotypes, *CES1* rs8192935 C/C or T/C genotype and *SLC19A1* rs1051266 G/G or G/A genotypes (see Table 4). Volunteers with *UGT1A6* rs10445704 A/A or *UGT2B7* rs7668258 T/T genotypes showed higher incidence of CNS symptoms compared to those with *UGT1A6* rs10445704 G/A or G/G genotype and *UGT2B7* rs7668258 T/C or C/C genotypes. Lastly, concerning MSADRs, volunteers with CYP2C19 PM phenotype showed an incidence of 50%, while both IM and NM phenotypes had 0% (see Table 4). The multivariate analysis of safety findings revealed no statistically significant association between exposure variables, sex or genetic variants, and ADR occurrence. No other association between genetic variants in other genes, including *5HT*, was associated with safety signals.

## 3. Discussion

Pregabalin is a drug with predictable pharmacokinetics, which can be attributed in part to its limited metabolism [9]. Since it is mainly excreted unaltered in urine, the main factor expected to explain interindividual differences in drug elimination would be renal function. Accordingly, the pregabalin drug label [1,5] warrants dose adjustments based on creatinine clearance values, which predict renal impairment status. However, due to the variability in drug response and tolerability, in this work, we hypothesized that genetic variation in some transporters could alter drug distribution or even elimination. As a secondary hypothesis, we proposed that patients with extreme pharmacogenetic phenotypes for certain metabolic enzymes could have a small alteration in pharmacokinetics. In our study, volunteers with renal function other than normal were excluded, which allowed us to control for this confounding factor and investigate the impact of other covariates on pregabalin pharmacokinetics.

The exposure metrics observed in this clinical trial agree with those reported in the literature and confirm the dose dependency or linearity of pregabalin over the range of doses used clinically. The pregabalin drug label [1,5] indicates an AUC_∞_ with the 150 mg dose of 28 µg*h/mL and a C_max_ of 3.8 µg/mL; here, for a dose of 300 mg, approximately a 2-fold increase in these metrics was observed (61 µg*h/mL and 7.8 µg/mL, respectively).

In this study, females showed a 1.3-fold higher exposure than males (for both exposure metrics, AUC and C_max_); however, after DW correction, the differences in the exposure metrics disappeared. The DW correction of exposure metrics captured the sex-associated differences related to individual size. Interestingly, males exhibited a higher V_d_/Fw, probably due to sex-specific differences in body composition. Moreover, pregabalin’s t_1/2_ was higher in males than in females. This finding seems contradictory based on the drug’s main elimination source, renal excretion [20], and may, therefore, be spurious based on the modest sample size.

Furthermore, several associations between genetic variants and pregabalin pharmacokinetics were observed. To our knowledge, no published reports support the hypothesis that pregabalin is a substrate of NAT2, UGT enzymes, or CYP enzymes. This may be explained by the fact that pregabalin is primarily excreted unchanged in urine and has not been specifically investigated in vivo, as suggested by preclinical studies. In this study, NAT2 SAs showed a 16–18% increase in exposure compared to IAs and NAs. The observed variations in t_1/2_ and Cl/F appear consistent, as NAT2 SAs exhibited a 25% higher t_1/2_ as compared with NAT2 IAs and 58% higher compared to NAT2 NAs. Our findings suggest that NAT2 could be partially responsible for the minor proportion of pregabalin metabolism. Additional studies are warranted to confirm if these differences in metabolism or exposure are clinically relevant.

Concerning the effect of *UGT1A6* rs10445704 and rs7592281, neither variant is considered actionable, nor has their functional impact been properly defined to date. This, combined with the lack of observed effect from the pharmacogenetic phenotype of UGT1A1, which does have a significant level of functional validation [21], suggests that these findings are not relevant and that pregabalin is unlikely to be a substrate of UGT enzymes. Moreover, only two or three volunteers were homozygous for the variant/variant genotypes, making it impossible to draw definitive conclusions on this matter.

A significant impact of *CES1* rs8192935 on C_max_ was observed (with the C allele associated with a higher C_max_ compared to the T allele), which was not observed for other metrics or parameters. The same occurred with CYP2B6 phenotype and t_1/2_, CYP3A5 phenotype and t_max_, *CYP4F2* diplotype and C_max_, *CYP1A2* rs2069526 and Cl/F, and *CYP2A6* rs28399433 and V_d_/F. No consistent differences were observed in the remaining parameters or metrics, suggesting that these findings may be random or spurious. In any case, the effect would be small, so they are not very relevant from a clinical point of view.

As described in the previous point, the metabolism of pregabalin could be partially affected by NAT2. However, the observed differences appeared to have no impact on tolerability, as no differences in ADR incidence were observed for this variable. In contrast, female sex appears to be the main factor predisposing to poorer tolerability, most likely due to the higher exposure observed in women compared to men. Consistently, ADR occurrence was linked to higher pregabalin concentrations. Although this study is based on single-dose administration in healthy volunteers, if this effect is confirmed in patients at a steady state, the possibility of dose adjustment based on sex, BMI, weight, or BSA should be considered.

The remaining associations identified as significant in the univariate analysis between genetic variants and safety findings may be considered random, which is likely attributable to the small sample size and multiple comparisons, thereby carrying a high risk of Type I error. This is consistent with the fact that they were no longer significant in the multivariate analysis. Therefore, it appears that only sex and its associated exposure are the primary determinants of pregabalin safety.

### Study Limitations

The main limitation of this study is the small sample size, which reduces the statistical power and the chances of encountering biomarkers of interest with low prevalence within the sample population. The absence of statistically significant differences in certain comparisons should be interpreted with caution (e.g., in the evaluation of the impact of biogeographic origin on pregabalin pharmacokinetics, only three European subjects were included) for the increased risk of a type II error (i.e., false negatives). It would be appropriate to increase the sample size in further confirmatory studies for greater statistical power and genetic variability. Nevertheless, a strength of this study is that it is the first to comprehensively analyze the relationship between genetic variants in clinically relevant pharmacogenes and the variability in pharmacokinetic parameters and exposure metrics of this drug. Moreover, our study was performed after a single-dose administration to healthy subjects, which does not allow for assessment of long-term effectiveness and safety. Pregabalin pharmacokinetics, pharmacodynamics, and tolerability might vary in patients with generalized anxiety disorder, epilepsy, or fibromyalgia. In contrast, the present work strictly controlled confounding factors that affect pharmacokinetics and safety (e.g., concomitant use of other drugs, smoking, etc.); therefore, the environment is more favorable than the clinical setting with patients.

## 4. Materials and Methods

### 4.1. Study Population

The study population comprised 24 healthy volunteers who participated in a bioequivalence clinical trial of pregabalin formulations conducted at the Clinical Trial Unit of Hospital Universitario de La Princesa (UECHUP). Each participant provided written informed consent twice—once for their involvement in the clinical trial and again for pharmacogenetic studies. The study protocols were reviewed and approved by the Hospital’s Research Ethics Committee and the Spanish Agency for Medicines and Medical Devices (AEMPS). In accordance with both Spanish and European regulations on human research, all procedures followed Good Clinical Practice guidelines and adhered to the principles outlined in the Declaration of Helsinki. The EUDRA-CT number was 2022-501160-18-00. The pharmacogenetic study was evaluated by the Independent Ethics Committee (IEC) of Hospital Universitario La Princesa and approved on 9 July 2020, with code SFC-FG-2020–1 (registration number: 4176).

Inclusion criteria were as follows: male or female subjects informed of the study’s details (objectives, risks, and right to withdraw) who gave written consent to participate, age 18–55 years, free from any psychiatric or organic conditions, normal medical records and physical examination, normal vital signs and electrocardiogram, and no clinically significant abnormalities in serology, hematology, coagulation, biochemistry, and urinalysis. Exclusion criteria comprised the following: affected by any organic or psychic condition, presenting clinically significant biochemical alterations in kidney and/or liver damage markers, BMI outside the 18.5–30 kg/m^2^ range, having received prescription drug treatment in the last 15 days or any kind of medication in the 48 h prior to receiving the study drug, except for contraceptive medication in women, history of hypersensitivity to any drug, positive drug screening (for cannabis, opiates, cocaine, and amphetamines), smokers, daily consumers of alcohol and/or acute alcohol poisoning in the last week, having donated blood in the previous month, pregnant or breastfeeding women, having participated in another clinical trial within the previous 3 months, swallowing difficulty, subjects with rare hereditary problems of galactose intolerance, lactase deficiency or glucose-galactose malabsorption and inability to follow the instructions or collaborate during the study.

### 4.2. Study Design and Procedures

The current observational pharmacogenetic study was based on a phase I bioequivalence clinical trial. It followed a crossover design, with two periods and two sequences, and the drug was administered under fasting conditions. All subjects received a 300 mg single dose of pregabalin (either the test or the reference formulation) orally around 9:00 am, with 240 mL of water, during the first study period. After a 7-day washout interval, they received the opposite formulation (period 2). The volunteers were hospitalized the day before each period (i.e., days −1 and 7) at around 10 pm until 9:30 pm on the dosing day (i.e., 12 h after drug administration on days 1 and 8).

During each study period, blood samples were collected from each volunteer via direct venipuncture or from an indwelling cannula in an arm vein. A total of 20 samples, each 3 mL in volume, were drawn into EDTA K2 tubes, starting from pre-dose and continuing up to 48 h after drug administration. After the extractions, the tubes were put into an ice-water bath and the person responsible for processing the samples transferred them to a centrifuge. The tubes were subsequently centrifuged at 4 °C during 10 min at 1900× *g*. Once centrifuged, 0.5 mL of plasma was aliquoted in tubes of polypropylene and stored in a freezer at −20 °C (±5 °C) until its shipment to an external analytical laboratory. The analytical method was validated according to EMA guidelines and involved a protein precipitation procedure with methanol. Pregabalin and internal standard were measured by reversed-phase high-performance liquid chromatography coupled to a tandem mass spectrometry detector (LC/MS/MS) with a lower limit of quantification (LLOQ) of 30 ng/mL.

### 4.3. Pharmacokinetics Analysis

Pharmacokinetic parameters were estimated using non-compartmental methods with the WinNonLin Professional Edition software (version 8.0, Scientific Consulting, Inc., Cary, NC, USA). Two parameters, t_max_ and C_max_, were directly derived from the raw data. AUC from zero to the last observed concentration time (t) (AUC_t_) was determined using the trapezoidal rule. The AUC from time t to infinity (AUC_t-inf_) was calculated by dividing the concentration at time t (C_t_) by the elimination constant (K_e_), which was obtained through linear regression of the log-linear phase of the plasma concentration curve. The total AUC (AUC_∞_) was calculated by adding AUC_t_ to AUC_t-inf_. The half-life (t_1/2_) was calculated as −ln2/K_e_. Additionally, pregabalin’s clearance and volume of distribution adjusted for bioavailability were determined as dose/AUC_∞_ and Cl/F divided by K_e_, respectively. Both were corrected by weight (Cl/Fw and V_d_/Fw). Pharmacokinetic parameters were analyzed as the mean values for both the T and R formulations for each volunteer since both formulations were bioequivalent.

### 4.4. Safety

The safety assessment involved evaluating abnormalities in analytical values, deriving from blood (i.e., biochemistry, coagulation, hematology, and serology) and urine (urinalysis, pregnancy test, drug abuse, and cotinine test) determinations, as well as physical examinations, vital signs (blood pressure and heart rate), serial electrocardiograms (ECGs), or any other clinically relevant events. Adverse events (AEs) reported either spontaneously by volunteers or in response to open-ended questions were also collected. Causality was determined using the Spanish Pharmacovigilance System algorithm [22], classifying AEs into five categories: definite, probable, possible, unlikely, and unrelated. Only definite, probable, or possible AEs were considered ADRs and included in the statistical analysis.

For the analysis of the ADRs, diarrhea, nausea, and vomiting were included in gastrointestinal symptoms; dizziness, drowsiness, and headache in CNS symptoms; and muscle spasms and muscle pain in MSADRs.

### 4.5. Genotyping, Haplotyping, and Phenotyping

One EDTA-K2 tube initially used for pharmacokinetic profiling was repurposed for genotyping. After plasma separation, the cell concentrate was resuspended in 0.9% NaCl to preserve the blood sample. DNA extraction was performed using the Maxwell^®^ RSC Instrument (Promega, Madison, WI, USA). Genotyping was carried out using a QuantStudio 12k Flex real-time PCR System (Applied Biosystems, ThermoFisher, Waltham, MA, USA), along with an OpenArray thermal block and a custom array. The method is based on quantitative polymerase chain reaction (qPCR) technology with allelic discrimination, utilizing TaqMan^®^ hydrolysis probes (Thermo Fisher Scientific, Waltham, MA, USA). Additionally, a copy number variation (CNV) assay was conducted on the same instrument, using a 96-well thermal block, to identify gene deletions (*5) or gene duplications (xN) for *CYP2D6* (assay IDs: Hs04083572_cn and Hs00010001_cn for intron 2 and exon 9, respectively; Thermo Fisher Scientific, Waltham, MA, USA), with RNAseP as the 2-copy reference [23]. Therefore, 114 variants in 31 genes were included in the present work (see Table 5). For simplicity, and in accordance with our specific context, genotyping-informed phenotyping was preferred over enzymatic studies to define the activity of transporters and enzymes.

Variant selection rationale: pharmacogenes encoding for transporters potentially affecting pregabalin distribution, including BBB permeation, absorption or elimination (e.g., *ABCB1*, *ABCG2* or *SLC* transporters) were considered primary candidate genes (Table 5). Pharmacogenes encoding for enzymes with well-defined phenotypic assignments were considered secondary candidates (e.g., *CYP2D6* or *CYP2C19*), as pregabalin reportedly undergoes minimal metabolism. Variants in serotonin (5-HT) receptors were included exploratorily for their possible effect on the tolerability of pregabalin, as well as in other enzymes or transporters without phenotypic assignment (Table 5).

Genotypes were used to determine haplotypes or alleles and to infer phenotypes when available. Allele definition was based on the core allele assignments from the Pharmacogene Variation Consortium (PharmVar) [24]. For functional assignments the Clinical Pharmacogenetics Implementation Consortium (CPIC) or Dutch Pharmacogenetis Working Group (DPWG) guidelines on different genes and drugs were used: *ABCG2*, *SLCOB1,* and statins [25]; *CYP2B6* and methadone [26]; *CYP2C19* and clopidogrel [27]; *CYP2C9* and nonsteroidal anti-inflammatory drugs [28]; *CYP2D6* and opioids [29]; *CYP3A4* and quetiapine [30]; *CYP3A5* and tacrolimus [31]; *CYP4F2* and warfarine [32]; and *UGT1A1* and atazanavir [21]. Another example is the involvement of NAT2 in the pharmacokinetics of rivaroxaban [33].

Variants in the remaining genes, which have no allelic or phenotypic assignments to date, were individually analyzed.

### 4.6. Statistical Analysis

Statistical analysis was performed using SPSS software (version 26.0, SPSS Inc., Chicago, IL, USA). Initially, a univariate analysis was conducted. Pharmacokinetic variables were logarithmically transformed to ensure normal distributions. To adjust for the influence of dose and weight on drug exposure, the AUC and C_max_ were normalized by dividing them by the DW ratio. For the comparison of means, a *t*-test was used for variables with two categories, while an ANOVA test followed by a Bonferroni post-hoc analysis was applied for variables with three or more categories. To compare the incidence of ADRs across categorical variables, either a Chi-squared test or Fisher’s exact test was employed to assess statistical significance in contingency tables. Subsequently, each pharmacokinetic parameter and ADR were analyzed individually using multivariate analysis with linear regression for pharmacokinetic parameters, as well as with logistic regression for ADRs. Categorical variables with more than two categories, such as variants, were converted into dummy variables. Additionally, any phenotype or genotype with a *p* value < 0.05 in the univariate analysis, along with race and sex (covariates), were included. For ADR multivariate analyses, pharmacokinetic parameters without DW correction were included as independent variables in the logistic regression. The Bonferroni correction was applied to account for multiple comparisons.

## 5. Conclusions

Sex primarily influenced pregabalin exposure, which is likely due to differences in body size, weight, and renal function between males and females. Pregabalin plasma levels are the main determinant of its tolerability. The NAT2 phenotype was associated with variations in pregabalin AUC and C_max_, suggesting that this enzyme may contribute to the drug’s minor metabolism. Nonetheless, the NAT2-related differences in exposure were small and did not affect pregabalin’s tolerability. Therefore, pharmacogenetic biomarkers appear to play a restrained role in pregabalin pharmacotherapy.

## Figures and Tables

**Table 1:** Demographic characteristics of the study population according to sex and race.

Variable		N	Age (Years)		Height (m)		Weight (kg)		BMI (kg/m^2^)	
			Mean	SD	Mean	SD	Mean	SD	Mean	SD
Sex	Female	14	33.21	9.96	1.57	0.05	59.05	6.51	23.85	2.52
	Male	10	34.60	6.64	1.74 *	0.05	80.02 *	10.35	26.37 *	3.02
Race	Mixed **	21	33.43	9.12	1.62	0.08	66.01	11.89	24.89	2.82
	European	3	36.33	2.08	1.79 *	0.08	80.23	10.01	24.92	4.59
Total		24	33.79	9.95	1.64	0.09	67.79	13.32	24.89	2.96

* *p* < 0.05 after *t*-test; ** Mixed refers to a population with 20 Latin Americans and one Sub-Saharan African. One volunteer only completed one of the clinical trial periods and did not consent to genotyping.

**Table 2:** Pharmacokinetic parameters according to sex and biogeographic group.

Variable		N	AUC_∞_/DW (kg*ng*h/mL*mg)		C_max_/DW (kg*ng/mL*mg)		t_max_ (h)		t _1/2_ (h)		V_d_/Fw (mL/kg)		Cl/Fw (mL/h·kg)	
			Mean	SD	Mean	SD	Mean	SD	Mean	SD	Mean	SD	Mean	SD
Sex	Female	14	13,230.17	2334.82	1685.27	252.37	1.83	0.76	5.21	0.84	572.88	68.96	77.83	13.16
	Male	10	13,656.04	2596.94	1764.10	354.46	1.45	0.36	6.53	0.79	704.43 *	90.95	75.76	14.35
Race	European	3	15,795.23	1814.36	1968.72	518.37	1.46	0.49	6.68	0.47	617.23	91.23	63.9	6.97
	Mixed	21	13,066.53	2307.53	1682.32	249.23	1.69	0.67	5.63	1.04	629.18	104.93	78.83	13.13
Total		24	13,407.61	2401.29	1718.12	294.52	1.67	0.64	5.76	1.04	43002.95	627.69	101.56	76.97

* *p* = 0.001 after *t*-test. Underlined: *p* < 0.05 after multivariate analysis (males vs. females; the dummy classification of genotypes included in this multivariate analysis is shown in Table 3 footnote). One volunteer only completed one of the clinical trial periods and did not consent to the pharmacogenetic study.

**Table 3:** Significant associations between genotypes or phenotypes and the pharmacokinetic parameters of pregabalin.

Genotype or Phenotype		N	AUC/DW (kg*ng*h/mL*mg)		C_max_/DW (kg*ng/mL*mg)		t_max_ (h)		t_1/2_ (h)		V_d_/Fw (mL/kg)		Cl/Fw (mL/h·kg)	
			Mean	SD	Mean	SD	Mean	SD	Mean	SD	Mean	SD	Mean	SD
*NAT2*	RA	2	12,443.09	2866.25	1929.11	94.95	1.92	0.83	4.32 *^1^	0.90	503.05	10.28	82.69	19.08
	IA	14	12,617.31	2075.98	1634.74	270.58	1.83	0.69	5.42 *^1^	0.81	628.01	97.49	81.21	12.52
	SA	7	14,687.88	2237.96	1785.95	351.79	1.41	0.36	6.80	0.63	680.38	89.14	69.65	10.43
*UGT1A6* rs7592281	G/G	20	12,820.75	1950.48	1643.39	232.28	1.72	0.62	5.74	1.10	648.81	97.81	79.84 *^2^	12.02
	G/T	2	17,675.61 *^2^	279.99	2250.64 *^2^	438.88	1.96	0.88	6.32	0.43	516.55	25.47	56.65	0.98
*CES1* rs8192935	T/T	2	11,710.92	607.46	1430.81	165.66	1.63	0.41	5.82	1.35	713.88	128.70	85.52	4.45
	T/C	12	13,317.30	2602.83	1616.04	243.28	2.00	0.70	5.80	1.06	637.08	94.51	77.92	15.25
	C/C	9	13,457.14	2109.56	1888.02 *^3^	294.43	1.34	0.31	5.66	1.15	609.79	105.01	75.98	11.20
*CYP2B6*	RM and NM	10	13,832.03	2507.47	1693.58	362.09	1.59	0.46	6.49 *^4^	0.86	688.82	89.36	74.58	13.34
	IM	10	13,174.82	2192.99	1712.34	228.35	1.92	0.78	5.39	0.79	598.78	96.99	77.96	13.18
	PM	3	11,425.06	1087.69	1729.03	364.61	1.42	0.46	4.45	0.66	561.61	56.94	88.19	8.24
*CYP3A5*	IM	6	13,480.36	2499.22	1638.82	289.55	2.33*^5^	0.73	5.67	1.10	611.94	102.93	76.39	13.98
	PM	17	13,144.80	2290.39	1730.20	302.33	1.49	0.42	5.77	1.08	640.54	101.42	78.33	13.08
*CYP4F2*	*1/*5	3	11,078.63	1185.08	1381.26	109.49	1.99	0.57	4.86	0.89	632.35	91.57	90.96	9.30
	*1/*1	6	12,765.53	1005.59	1627.38	189.06	1.83	0.92	5.41	0.65	614.55	79.98	78.90	6.24
	*1/*3, *1/*4	3	13,719.11	3800.88	2186.91 *^6^	323.96	1.33	0.00	5.81	1.86	615.04	160.86	76.65	20.14
	*1/*6	3	12,948.61	2532.90	1765.61	357.46	1.82	0.61	5.49	0.64	627.77	134.12	79.57	17.49
	*1/*2, *2/*2	2	16,231.10	1762.85	1768.88	242.42	2.29	0.41	6.13	0.16	550.28	73.17	62.07	6.68
	*3/*6, *4/*6	2	14,839.29	3218.11	1713.27	252.91	1.58	0.76	6.38	2.28	617.42	99.41	69.38	14.58
	*3/*3, *3/*4, *4/*4	3	13,410.05	2449.35	1656.15	121.29	1.29	0.23	6.46	0.81	702.59	63.71	76.55	15.46

*^1^ *p* < 0.05 after ANOVA and Bonferroni post-hoc, RA vs. SA; IA vs. SA; *^2^ *p* < 0.05 after *t*-test; *^3^ *p* < 0.05 vs. T/C after ANOVA and Bonferroni post-hoc; *^4^ *p* < 0.05 vs. IM and PM after ANOVA and Bonferroni post-hoc; *^5^ *p* <0.05 after *t*-test; *^6^ *p* < 0.05 vs. *1/*5 after ANOVA and Bonferroni post-hoc; underlined *p* < 0.05 after multivariate analysis (males vs. females; NAT2 SA vs. RA and IA; *CES1* rs8192935 C/C vs. T/T and T/C; *CYP3A5* IM vs. PM; CYP4F2 *1/*1 and *1/*5 vs. the rest). RA: rapid acetylator; IA: intermediate acetylator; SA: slow acetylator; RM: rapid metabolizer; NM: normal metabolizer; IM: intermediate metabolizer; PM: poor metabolizer.

**Table 4:** Nominally significant associations between sex, genetic variants, or genotype-informed phenotypes and the incidence of adverse drug reactions.

Adverse Drug Reaction	Variable		Prevalence	*p*
Any	Sex	Female	14 out of 14 (100%)	*p* = 0.006
		Male	5 out of 10 (50%)	
Any	*ABCB1* rs2032582	G/G	6 out of 6 (100%)	*p* = 0.040
		G/T	10 out of 12 (83.3%)	
		T/T	2 out of 5 (40%)	
GI symptoms	*CES1* rs2244613	C/C	2 out of 2 (100%)	*p* = 0.042
		C/A	1 out of 7 (14.3%)	
		A/A	2 out of 14 (14.3%)	
GI symptoms	*CES1* rs8192935	T/T	2 out of 2 (100%)	*p* = 0.026
		T/C	1 out of 12 (8.3%)	
		C/C	7 out of 9 (77.8%)	
GI symptoms	*SLC19A1* rs1051266	G/G	2 out of 8 (25%)	*p* = 0.020
		G/A	0 out of 10 (0%)	
		A/A	3 out of 5 (60%)	
CNS symptoms	Sex	Female	13 out of 14 (92.9%)	*p* = 0.050
		Male	5 out of 10 (50%)	
CNS symptoms	*UGT1A6* rs10445704	G/G	4 out of 9 (44.4%)	*p* = 0.038
		G/A	10 out of 11 (90.9%)	
		A/A	3 out of 3 (100%)	
CNS symptoms	*UGT2B7* rs7668258	T/T	5 out of 5 (100%)	*p* = 0.031
		T/C	10 out of 12 (83.3%)	
		C/C	2 out of 6 (33.3%)	
MSADR	CYP2C19	NM	0 out of 5 (0.0%)	*p* = 0.026
		IM	0 out of 13 (0.0%)	
		PM	2 out of 4 (50%)	

*p* value: univariate analysis. GI: gastrointestinal; CNS: central nervous system; MSADR: musculoskeletal adverse drug reactions; NM: normal metabolizer; IM: intermediate metabolizer; PM: poor metabolizer.

**Table 5:** Genes, alleles, and genotyped variants (rs numbers).

Gene	Allele Name	Variants
*5HT1A*	N/A	rs6295 (C>G)
*5HT2A*	N/A	rs6311 (G>A)
	N/A	rs6314 (C>T)
	N/A	rs7997012 (T>C)
*ABCB1*	Legacy name: C3435T	rs1045642 (T>C)
	Legacy name: C1236T	rs1128503 (T>C)
	Legacy name: G2677 T/A	rs2032582 (T>G/A)
*ABCC2*	N/A	rs2273697 (G>A)
	N/A	rs3740066 (C>T)
*ABCC3*	N/A	rs4793665 (C>T)
*ABCG2*	N/A	rs2231142 (C>A)
*CES1*	N/A	rs2244613 (C>A)
	N/A	rs71647871 (G>A)
	N/A	rs8192935 (T>C)
*CYP1A2*	N/A	rs2470890 (T>C)
	N/A	rs2069514 (G>A)
	N/A	rs2069526 (T>G)
	N/A	rs762551 (C>A)
	N/A	rs12720461 (C>T)
	N/A	rs72547516 (A>G)
*CYP2A6*	N/A	rs28399433 (T>G)
*CYP2B6*	*4	rs2279343 (A>G)
	*5	rs3211371 (C>T)
	*6	rs3745274 (G>T), rs2279343 (A>G)
	*7	rs3745274 (G>T), rs2279343 (A>G), rs3211371 (C>T)
	*9	rs3745274 (G>T)
	*22	rs34223104 (T>C)
*CYP2C19*	*2	rs4244285 (G>A), rs12769205 (A>G)
	*3	rs4986893 (G>A)
	*4	rs28399504 (A>G)
	*5	rs56337013 (C>T)
	*6	rs72552267 (G>A)
	*7	rs72558186 (T>A)
	*8	rs41291556 (T>C)
	*9	rs17884712 (G>A)
	*17	rs12248560 (C>T)
	*35	rs12769205 (A>G)
*CYP2C8*	*2	rs11572103 (A>G)
	*3	rs10509681 (A>G), rs11572080 (G>A)
	*4	rs1058930 (C>G)
*CYP2C9*	*2	rs1799853 (C>T)
	*3	rs1057910 (A>C)
	*5	rs28371686 (C>G)
	*6	rs9332131 (delA)
	*8	rs7900194 (G>A)
	*11	rs28371685 (C>T)
	*45	rs199523631 (C>T)
*CYP2D6*	*2	rs16947 (C>T), rs1135840 (G>C)
	*3	rs35742686 (T>delT)
	*4	rs3892097 (G>A), rs1065852 (C>T), rs1135840 (G>C)
	*6	rs5030655 (A>delA)
	*7	rs5030867 (T>G)
	*8	rs5030865 (G>A), rs16947 (C>T), rs1135840 (G>C)
	*9	rs5030656 (AAG>delAAG)
	*10	rs1065852, rs1135840 (G>C)
	*12	rs5030862 (G>A), rs16947 (C>T), rs1135840 (G>C)
	*14	rs5030865 (G>A), rs16947 (C>T), rs1135840 (G>C)
	*15	rs774671100 (A>dupA)
	*17	rs28371706 (C>T), rs16947 (C>T), rs1135840 (G>C)
	*19	rs72549353 (AACT>delAACT)
	*29	rs59421388 (G>A), rs1135840 (G>C), rs16947 (C>T)
	*41	rs28371725 (G>A), rs1135840 (G>C), rs16947 (C>T)
	*56	rs72549347 (C>T)
	*59	rs79292917 (G>A)
*CYP3A4*	*2	rs55785340 (T>C)
	*3	rs4986910 (T>C)
	*4	rs55951658 (A>G)
	*5	rs55901263 (C>G)
	*6	rs4646438 (A>dupA)
	*18	rs28371759 (T>C)
	*20	rs67666821 (T>TT)
	*22	rs35599367 (C>T)
	*36	rs2242480 (G>A)
	N/A	rs2740574 (G>A)
*CYP3A5*	*3	rs776746 (A>G)
	*6	rs10264272 (G>A)
	*7	rs41303343 (T>dupT)
*CYP4F2*	*7	rs114099324 (G>C)
	*3 *4	rs2108622 (G>A)
	*2 *4	rs3093105 (T>G)
	*5	rs3093200 (C>A)
	*6	rs3093153 (G>T)
*NAT2*	*5	rs1801280 (T>C)
	*6	rs1799930 (G>A)
	*7	rs1799931 (G>A)
*SLC19A1*	N/A	rs1051266 (T>C)
*SLC22A1*	N/A	rs12208357 (C>T)
	N/A	rs34059508 (G>A)
	N/A	rs628031 (A>G)
	N/A	rs72552763 (GAT>delGAT)
*SLC22A2*	N/A	rs316019 (A>C)
*SLC6A2*	N/A	rs12708954 (C>A)
	N/A	rs3785143 (C>T)
*SLCO1B1*	*4	rs1104581 (C>A)
	*14	rs2306283 (A>G), rs11045819 (C>A)
	*19	rs34671512 (A>C)
	*23	rs373327528 (G>A)
	*5	rs4149056 (T>C)
	*9	rs59502379 (G>C)
*UGT1A1*	N/A	rs4148323 (G>A)
	N/A	rs887829 (C>T)
*UGT1A3*	N/A	rs2008584 (A>G)
*UGT1A4*	N/A	rs2011425 (T>G)
*UGT1A6*	N/A	rs10445704 (G>A)
	N/A	rs7592281(G>T)
*UGT1A8*	N/A	rs1042597 (C>T)
*UGT2B10*	N/A	rs61750900 (G>T)
*UGT2B15*	N/A	rs1902023 (A>C)
*UGT2B7*	N/A	rs7668258 (T>C)

## Data Availability

The original contributions presented in the study are included in the article, further inquiries can be directed to the corresponding authors.
